# Development and validation of an analysis method for pesticide residues by gas chromatography–tandem mass spectrometry in Daikenchuto

**DOI:** 10.1007/s11418-020-01473-y

**Published:** 2021-01-03

**Authors:** Hirokazu Saegusa, Hiroshi Nomura, Masaki Takao, Takashi Hamaguchi, Masaru Yoshida, Yuzo Kodama

**Affiliations:** 1 Division of Gastroenterology, Department of Internal Medicine, Kobe University Graduate School of Medicine, 7-5-1 Kusunoki-Cho, Chuo-ku, Kobe, Hyogo 650-0017 Japan; 2 CMC Research and Development Laboratories, Tsumura and Co., 3586 Yoshiwara, Ami-machi, Inashiki-gun, Ibaraki, 300-1192 Japan; 3 Division of Metabolomics Research, Department of Internal Medicine Related, Kobe University Graduate School Medicine, 7-5-1 Kusunoki-Cho, Chuo-ku, Kobe, Hyogo 650-0017 Japan

**Keywords:** Herbal medicine, U.S. pharmacopeia, Matrix effect, Organophosphorous pesticides

## Abstract

Daikenchuto (DKT) is one of the most widely used “Kampo” in Japan as a representative of herbal medicine. Because DKT is made from a natural product like food, it requires the management of pesticides; therefore, an analysis of residual pesticides in Kampo is required. The World Health Organization (WHO) indicates that pesticide residue analysis by the U.S. Pharmacopeia (USP) is required. USP defines 107 compounds containing organochlorine pesticides and organophosphorus pesticides and their metabolites, which have a high residual risk. Accordingly, to guarantee the safety of herbal medicines according to global standards is a very important issue. In this study, we developed an analytical method for 91 compounds, which are listed in USP, using DKT as the subject. The method could extract pesticides from DKT with acetone, elute pesticides with acetonitrile using a SepPak C18 column (5 g) and with ethyl acetate using a DSC-NH_2_ column (2 g), and perform simultaneous analyses by gas chromatography–tandem mass spectrometry (GC–MS/MS). This method, which could quantify 88 compounds, was validated according to USP. A pesticide residue analysis method that meets USP requirements enables the analysis of pesticide residues with a high residue risk and contributes to improving the safety of “Kampo” and other herbal medicines.

## Introduction

Since ancient times, medicinal plants have been employed globally for treatments, and some have been systematized and classified as traditional medicines [1]. Japanese traditional herbal medicine (Kampo) originated from ancient Chinese medicine before it was introduced in Japan around the fifth century. Thereafter, Kampo has become one of the herbal medicines that have accomplished independent development in the country [2, 3]. Daikenchuto (DKT) is a Kampo that is prepared from three herbal materials, i.e., ginseng, ginger, and Japanese pepper. It is largely utilized in Japan. Daikenchuto has been employed in the improvement of various symptoms of the lower abdominal region, such as bloating, abdominal pain, and constipation [3–5]. In recent years, many clinical study results of DKT regarding digestive tract symptoms in humans have been reported, and an elucidation of its mechanism in gastrointestinal hyperactivity is in progress [3–5]. It is expected that DKT will be widely explored in the medical field in the future. Conversely, there is strong demand to secure the safety of medicinal plants themselves and the drugs that are prepared from them because of their usefulness and extensive application [1]. 

Pesticides, such as microbicides and insecticides, are widely employed in the cultivations of medicinal plants and other farm products. They are essential in ensuring the stable quantity and quality of plants. However, contamination due to pesticide residues is generally a problem. Generally, pesticide residue standards in the food sector of each country are set, based on the acceptable daily intake [1, 6]. Moreover, regarding medicinal plants, the recommendations of WHO follow the Japanese Pharmacopeia (JP), US Pharmacopeia (USP), and European Pharmacopoeia (EP) [7–9]. US Pharmacopeia and EP target 70 items, including persistently stable organophosphorus and organochlorine pesticides [8, 9].

The 70 items set by USP include major compounds (metabolites and isomers), and a total of 107 compounds are the subject of analysis. Since the 107 compounds are mostly low to medium polar compounds, gas chromatography–tandem mass spectrometry (GC–MS/MS) analysis is required. In the GC–MS/MS analysis, highly polar substances are known to affect the process. Therefore, it is necessary to remove highly polar substances, employing a SPE (solid-phase extraction) column. In the past, the analyses of USP-listed pesticides by GC–MS/MS have been reported in terms of botanical drug substances. One such report was on “Ginseng” where 62 out of the 107 compounds were eluted by a mixed solution of acetone and toluene in a PSA column [10]. The other is a report on "Chenpi" where 67 out of the 107 compounds were purified by a dispersed solid phase [11]. In these reports, since only a few compounds could be analyzed, it became a challenge to expand the number of pesticides that could be analyzed by GC–MS/MS. The purpose of this study is to develop a pretreatment method for 91 USP-listed compounds that can be analyzed by GC–MS/MS, in DKT. Thus far, there has been no report on the development of an analysis method for USP-listed compounds, in DKT. Since DKT contains a wide variety of ingredients, which are derived from Ginseng, ginger, and Japanese pepper, an analysis method for individual raw materials cannot be employed. In this method, to develop a new pretreatment method for DKT, a purification method utilizing ethyl acetate, which is less polar than acetone, as an elution solvent was used. Additionally, the developed analytical method was validated according to USP.

## Experimental

### Chemicals and other materials

Standard reagents of pesticides were purchased, as shown in Table 1. Acetone (pesticide residue and polychlorinated biphenyl (PCB) analysis grade), acetonitrile (pesticide residue and PCB analysis grade), ethyl acetate (pesticide residue and PCB analysis grade), sodium chloride (guaranteed reagent), and formic acid (high-performance liquid chromatography (HPLC) grade) were purchased from Kanto Chemical Corporation (Tokyo, Japan). Anhydrous sodium sulfate (pesticide residue and PCB analysis grade), methanol (HPLC grade), and 1 mol/L ammonium formate solution (HPLC grade) were purchased from FUJIFILM Wako Pure Chemical Corporation (Osaka, Japan). SepPak C18 (5 g) was purchased from Waters (MA, USA). Discovery DSC-NH_2_ (2 g), Discovery DSC-Si (5 g), and l-gulonic acid γ-lactone were purchased from Sigma-Aldrich (MO, USA). Mega Bond Elut PSA (2 g) was purchased from Agilent Technologies (CA, USA).

**Table 1:** Data of USP standard values, suppliers, and MRM conditions of selected pesticides

USP		Compounds	Supplier	Analytical concentration (μg/mL)	MRM conditions			
Pesticides	MRL (mg/kg)				MRM1	CE^a^	MRM2	CE^a^
Alachlor	0.05	Alachlor	W	0.05	188.0 → 160.0	8	188.0 → 132.0	18
Aldrin and dieldrin (sum of)	0.05	Aldrin	D	0.05	293.0 → 220.0	24	293.0 → 258.0	12
		Dieldrin	A	0.05	263.0 → 193.0	28	277.0 → 241.0	8
Azinphos-ethyl	0.1	Azinphos-ethyl	D	0.1	160.0 → 132.0	2	132.0 → 77.0	14
Azinphos-methyl	1	Azinphos-methyl	W	1	160.0 → 132.0	2	132.0 → 77.0	14
Bromophos-ethyl	0.05	Bromophos-ethyl	D	0.05	359.0 → 331.0	6	303.0 → 239.0	22
Bromophos-methyl	0.05	Bromophos-methyl	D	0.05	331.0 → 316.0	18	329.0 → 314.0	18
Bromopropylate	3	Bromopropylate	K	3	341.0 → 183.0	18	341.0 → 185.0	18
Chlordane (sum of *cis-*, *trans-*, and oxychlordane)	0.05	*cis-*Chlordane	D	0.05	373.0 → 266.0	22	375.0 → 266.0	22
		*trans-*Chlordane	D	0.05	373.0 → 266.0	22	375.0 → 266.0	22
		Oxychlordane	A	0.05	389.0 → 353.0	6	387.0 → 323.0	14
Chlorfenvinphos	0.5	*cis-*Chlorfenvinphos	W	0.5	267.0 → 159.0	16	323.0 → 267.0	14
		*trans-*Chlorfenvinphos	H	0.5	267.0 → 159.0	16	323.0 → 267.0	14
Chlorpyriphos-ethyl	0.2	Chlorpyriphos-ethyl	W	0.2	314.0 → 258.0	14	286.0 → 258.0	6
Chlorpyriphos-methyl	0.1	Chlorpyriphos-methyl	W	0.1	286.0 → 93.0	24	288.0 → 93.0	24
Chlorthal-dimethyl	0.01	Chlorthal-dimethyl	D	0.01	301.0 → 223.0	26	301.0 → 273.0	16
Cyfluthrin (sum of)	0.1	Cyfluthrin	D	0.1	226.0 → 206.0	14	226.0 → 199.0	6
*λ-*Cyhalothrin	1	Cyhalothrin	D	1	208.0 → 181.0	6	197.0 → 141.0	14
Cypermethrin and isomers (sum of)	1	Cypermethrin	W	1	181.0 → 152.0	26	209.0 → 116.0	14
DDT (sum of *o,p'-*DDE, *p,p'-*DDE, *o,p'-*DDD, *p,p'-*DDD, *o,p'-*DDT, *p,p'-*DDT)	1	*o,p'-*DDE	A	1	246.0 → 176.0	28	318.0 → 248.0	22
		*p,p'-*DDE	W	1	246.0 → 176.0	28	318.0 → 248.0	22
		*o,p'-*DDD	D	1	235.0 → 165.0	24	237.0 → 165.0	24
		*p,p'-*DDD	W	1	235.0 → 165.0	24	237.0 → 165.0	24
		*o,p'-*DDT	D	1	235.0 → 165.0	24	237.0 → 165.0	24
		*p,p'-*DDT	W	1	235.0 → 165.0	24	237.0 → 165.0	24
Deltamethrin	0.5	Deltamethrin	W	0.5	253.0 → 93.0	18	253.0 → 174.0	6
Diazinon	0.5	Diazinon	W	0.5	304.0 → 179.0	8	179.0 → 137.0	20
Dicofol	0.5	Dicofol (4,4′-dichlorobenzophenone)	D	0.5	250.0 → 215.0	4	252.0 → 141.0	12
Endosulfan (sum of isomers and endsulfan-sulfate)	3	*α-*Endosulfan	W	3	265.0 → 194.0	8	339.0 → 267.0	4
		*β-*Endosulfan	D	3	265.0 → 194.0	10	339.0 → 267.0	4
		Endosulfan-sulfate	D	3	272.0 → 237.0	14	387.0 → 253.0	10
Endrin	0.05	Endrin	D	0.05	281.0 → 245.0	10	281.0 → 209.0	22
Ethion	2	Ethion	W	2	231.0 → 175.0	12	231.0 → 203.0	6
Etrimphos	0.05	Etrimphos	D	0.05	292.0 → 181.0	6	292.0 → 153.0	22
Fenchlorphos (sum of fenchlorphos and fenchlorphos oxon)	0.1	Fenchlorphos	D	0.1	287.0 → 272.0	16	285.0 → 240.0	28
		Fenchlorphos oxon	D	0.1	269.0 → 254.0	18	269.0 → 224.0	28
Fenitrothion	0.5	Fenitrothion	W	0.5	277.0 → 260.0	4	277.0 → 109.0	18
Fenpropathrin	0.03	Fenpropathrin	W	0.03	265.0 → 210.0	10	265.0 → 89.0	28
Fensulfothion (sum of fensulfothion, fensulfothion oxon, fensulfothion-oxon-sulfone and fensulfothion-sulfone)	0.05	Fensulfothion	W	0.05	308.0 → 293.0	4	293.0 → 125.0	12
		Fensulfothion oxon	D	0.05	277.0 → 249.0	6	277.0 → 221.0	12
		Fensulfothion-oxon-sulfone	D	0.05	308.0 → 182.0	4	293.0 → 229.0	4
		Fensulfothion-sulfone	D	0.05	324.0 → 170.0	4	296.0 → 188.0	8
Fenthion (sum of fenthion, fenthion-oxon, fenthion-oxon-sulfone, fenthion-oxon-sulfoxide, fenthion-sulfone and fenthion sulfoxide)	0.05	Fenthion	D	0.05	278.0 → 109.0	20	278.0 → 125.0	20
		Fenthion oxon	W	0.05	262.0 → 247.0	10	262.0 → 217.0	16
		Fenthion-oxon-sulfone	W	0.05	215.0 → 109.0	12	294.0 → 230.0	6
		Fenthion-oxon-sulfoxide	W	0.05	263.0 → 109.0	16	278.0 → 263.0	6
		Fenthion-sulfone	D	0.05	310.0 → 246.0	4	310.0 → 136.0	18
		Fenthion sulfoxide	D	0.05	294.0 → 279.0	4	279.0 → 169.0	14
Fenvalerate	1.5	Fenvalerate	W	1.5	225.0 → 119.0	18	225.0 → 147.0	8
Flucythrinate	0.05	Flucythrinate	W	0.05	451.0 → 199.0	10	225.0 → 147.0	8
τ-Fluvalinate	0.05	Fluvalinate	W	0.05	250.0 → 55.0	16	250.0 → 200.0	22
Fonophos	0.05	Fonophos	W	0.05	246.0 → 109.0	16	246.0 → 137.0	4
Heptachlor (sum of heptachlor, *cis-*heptachlorepoxide and *trans-*heptachlorepoxide)	0.05	Heptachlor	D	0.05	272.0 → 237.0	16	337.0 → 266.0	16
		*cis-*Heptachlorepoxide	D	0.05	353.0 → 263.0	14	355.0 → 265.0	16
		*trans-*Heptachlorepoxide	D	0.05	353.0 → 253.0	16	355.0 → 291.0	8
Hexachlorbenzene	0.1	Hexachlorobenzene	D	0.1	284.0 → 249.0	22	249.0 → 214.0	16
Hexachlorcyclohexane (sum of isomers *α-*, *β-*, *δ-* and *ε-*)	0.3	*α-*Hexachlorcyclohexane	D	0.3	219.0 → 183.0	6	181.0 → 145.0	14
		*β-*Hexachlorcyclohexane	D	0.3	219.0 → 183.0	8	181.0 → 145.0	16
		*δ-*Hexachlorcyclohexane	D	0.3	219.0 → 183.0	8	181.0 → 145.0	16
		*ε-*Hexachlorcyclohexane	K	0.3	219.0 → 183.0	8	181.0 → 145.0	14
Lindane (*γ-*hexachlorocychlohexane)	0.6	*γ-*Hexachlorcyclohexane	W	0.6	219.0 → 183.0	6	181.0 → 145.0	14
Malathion and malaoxon (sum of)	1	Malathion	D	1	173.0 → 99.0	14	173.0 → 127.0	4
		Malaoxon	W	1	268.0 → 127.0	6	268.0 → 99.0	12
Mecarbam	0.05	Mecarbam	D	0.05	329.0 → 131.0	14	296.0 → 196.0	6
Methacriphos	0.05	Methacriphos	D	0.05	240.0 → 208.0	2	208.0 → 180.0	4
Methidathion	0.2	Methidathion	D	0.2	145.0 → 85.0	6	145.0 → 58.0	14
Methoxychlor	0.05	Methoxychlor	W	0.05	227.0 → 169.0	28	227.0 → 212.0	16
Mirex	0.01	Mirex	D	0.01	272.0 → 237.0	16	332.0 → 297.0	22
Parathion-ethyl and paraoxon-ethyl (sum of)	0.5	Parathion-ethyl	W	0.5	291.0 → 109.0	12	291.0 → 81.0	28
		Paraoxon-ethyl	D	0.5	275.0 → 99.0	16	275.0 → 149.0	4
Parathion-methyl and paraoxon-methyl (sum of)	0.2	Parathion-methyl	W	0.2	263.0 → 109.0	10	246.0 → 216.0	4
		Paraoxon-methyl	F	0.2	230.0 → 200.0	6	230.0 → 136.0	10
Pentachloranisole	0.01	Pentachloroanisole	D	0.01	265.0 → 237.0	14	280.0 → 237.0	26
Permethrin and isomers (sum of)	1	*cis-*Permethrin	D	1	183.0 → 168.0	14	163.0 → 127.0	6
		*trans-*Permethrin	W	1	183.0 → 168.0	14	163.0 → 127.0	6
Phosalone	0.1	Phosalone	W	0.1	182.0 → 111.0	16	367.0 → 182.0	6
Phosmet	0.05	Phosmet	W	0.05	160.0 → 133.0	14	317.0 → 160.0	4
Pirimiphos-ethyl	0.05	Pirimiphos-ethyl	D	0.05	318.0 → 166.0	14	333.0 → 180.0	6
Pirimiphos-methyl(sum of pirimiphos-methyl and *N-*desethyl-pirimiphos-methyl)	4	Pirimiphos-methyl	D	4	290.0 → 233.0	10	305.0 → 290.0	8
		*N-*desethyl-pirimiphos-methy	D	4	277.0 → 168.0	6	277.0 → 262.0	8
Procymidone	0.1	Procymidone	D	0.1	283.0 → 96.0	10	283.0 → 255.0	10
Profenophos	0.1	Profenophos	W	0.1	339.0 → 269.0	14	339.0 → 311.0	4
Prothiophos	0.05	Prothiophos	W	0.05	267.0 → 239.0	8	309.0 → 239.0	16
Quinalphos	0.05	Quinalphos	D	0.05	298.0 → 156.0	6	270.0 → 189.0	8
Quintozene (sum of quintozene, pentachloroaniline and methylpentachlorophenyl sulfide)	1	Quintozene	W	1	295.0 → 237.0	18	295.0 → 265.0	8
		Pentachloroaniline	W	1	265.0 → 194.0	24	267.0 → 194.0	24
		Methyl Pentachlorophenyl sulfide	F	1	296.0 → 263.0	16	296.0 → 246.0	28
s-421	0.02	s-421	D	0.02	211.0 → 79.0	6	181.0 → 85.0	8
Tecnazene	0.05	Tecnazene	W	0.05	261.0 → 203.0	14	215.0 → 179.0	10
Tetradifon	0.3	Tetradifon	W	0.3	227.0 → 199.0	16	356.0 → 159.0	12
Vinclozolin	0.4	Vinclozolin	D	0.4	285.0 → 212.0	12	212.0 → 172.0	14

^a^Collision energy

MRL = maximum residue limit

*A* AccuStandard Inc. (New Haven, USA), *D* Dr. Ehrenstorfer (Augsburg, Germany), *F* Fluka Biochemika (Buchs, Switzerland), *H* Hayashi pure chemical (Osaka, Japan), *K* Kanto Chemical (Tokyo, Japan), *W* FUJIFILM Wako Pure Chemical Corporation (Osaka, Japan)

### Preparation of pesticide standard solutions

Each pesticide standard reagent, shown in Table 1, was dissolved in acetone to prepare a 1 mg/ml stock solution. The respective stock solutions were mixed to prepare a standard solution for addition (four times the analytical concentration). Thereafter, the standard solution for the addition was diluted with acetone to prepare a standard solution with analytical concentration.

### Instruments

Gas chromatography–tandem mass spectrometry (Agilent Technologies 7890GC/7000B, CA, USA) was employed. A DB-XLB (length 30 m, thickness 0.25 µm, and inner diameter 0.25 mm) capillary column (Agilent Technologies, CA, USA) was employed for the separation. The injection mode was pulsed splitless, its volume was 2.0 µL, and its temperature was 280 °C [12]. The temperature program of the oven was set at 80 °C for 2 min, ramped 20 °/min to 200 °C, ramped 10 °C /min to 300 °C, hold at 300 °C for 5 min, ramped 25 °C/min to 325 °C, and held at 325 °C for 11 min. Further, electron ionization (EI) and multiple reaction monitoring (MRM) modes were employed for quantitation. The EI voltage was 70 eV, and the MRM conditions are given in Table 1. MRM is a combination of the precursor and productions. The compound was quantified by MRM1, employing the peak area value. The ratio of the peak area value of MRM2 to that of MRM1 was qualitatively compared to the standard solution.

### Sample treatment and preparation

#### Evaluation of the C18 SPE column purification process with a standard solution

A standard solution or acetonitrile (20 mL) was added to the SepPak C18 (5 g) columns to activate them. Thereafter, 40 mL of acetonitrile was added, and the eluent was collected in a flask. Afterward, 40 mL of acetone was added for the elution, and the eluent was collected in another flask. The eluent in the flask was concentrated and dissolved in acetone for the GC–MS/MS measurement.

#### Liquid–liquid distribution and the dehydration process with the standard solution

The standard solution, 10 mL of acetonitrile, 10 mL of saturated sodium chloride solution, and 25 mL of ethyl acetate were mixed in a centrifuge tube. The mixture was shaken and the upper layer was collected in a new flask. Anhydrous sodium sulfate was added, and the mixture was filtrated. Further, 25 mL of ethyl acetate was added and shaken. The upper layer was collected in the flask and was dehydrated by anhydrous sodium sulfate.

#### Purification column tests

The standard solutions and 20 mL of ethyl acetate were added to each purification columns that had been activated with ethyl acetate. Next, 20 mL of ethyl acetate was added, and the eluent was collected in a new flask. The eluent was concentrated and dissolved in acetone for the GC–MS/MS measurement.

#### Recovery test

**Fig. 1:**
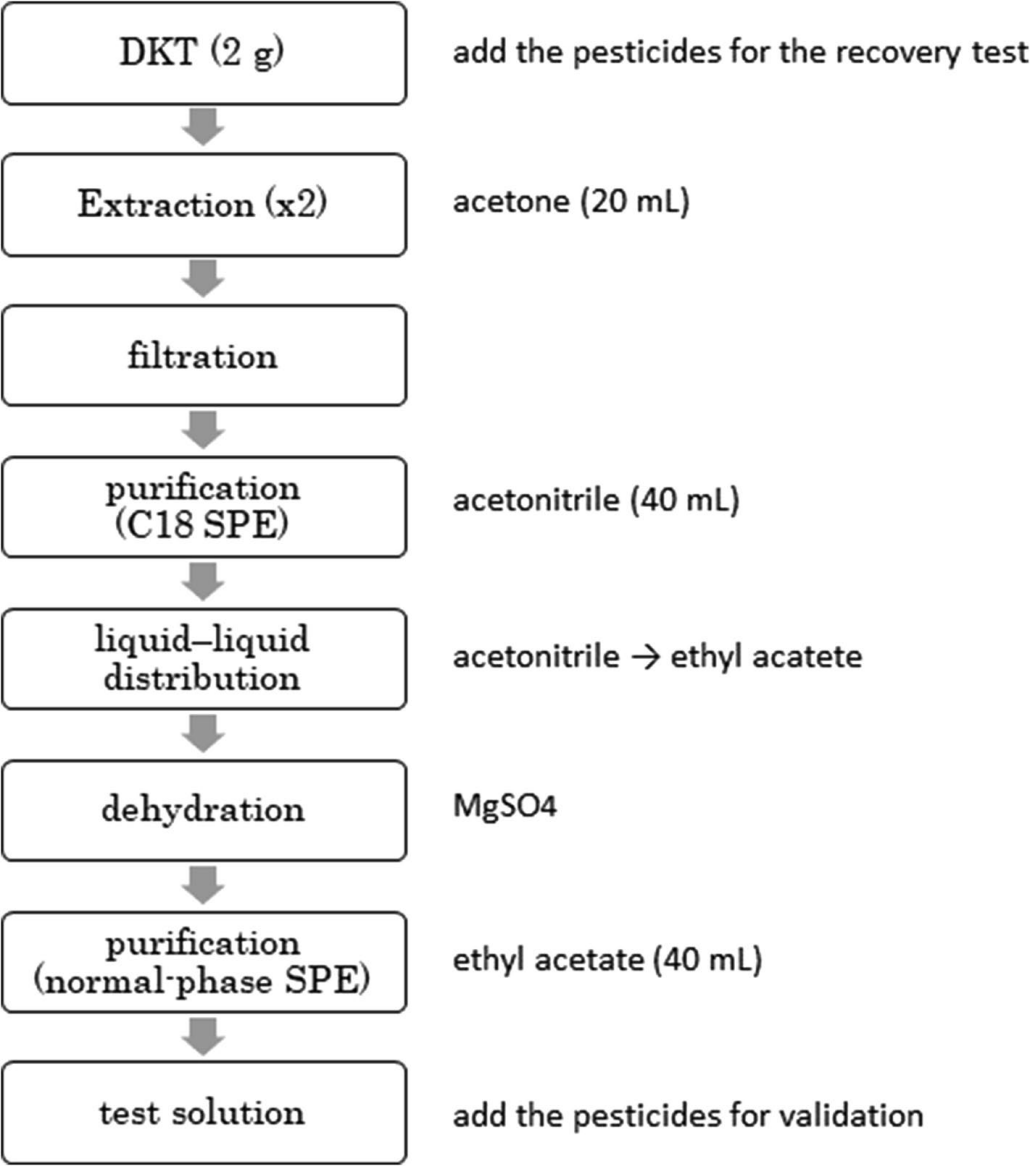
Sample preparation flow

The recovery test in DKT was performed, as shown in Fig. 1. The detailed steps are given as follows: DKT was crushed with a mortar and pestle, a 2 g portion, which was placed in a centrifuge tube, was accurately weighed. For the recovery test, the standard solutions were added to the centrifuge tube, after which 20 mL of acetone was added to the tube; shaken for 10 min, at 200 rpm; and centrifuged for 5 min, at 3,000 rpm. The supernatant was collected in a new flask, and 20 mL of acetone was placed in the centrifuge tube, shaken for 10 min, at 200 rpm, and centrifuged for 5 min, at 3,000 rpm. The supernatant obtained from the second extraction was mixed with that from the first; concentrated, at reduced pressure; and redissolved in 20 mL of acetonitrile. The acetonitrile solution was applied to a SepPak C18 (5 g) solid-phase extraction column, which had been activated with acetonitrile, and the eluate was collected in a flask. Further, 40 mL of acetonitrile was applied to the SepPak C18 (5 g) column, and the eluate was collected in the same flask. This eluate was concentrated, at reduced pressure; transferred into a centrifuge tube; and 10 mL of saturated sodium chloride solution and 25 mL of ethyl acetate were added. The mixture was shaken for 10 min, at 200 rpm; centrifuged for 5 min, at 3,000 rpm; and the upper layer was transferred into a new flask. Next, 25 mL of ethyl acetate was added again, and the mixture was shaken for 10 min, at 200 rpm; centrifuged for 5 min, at 3,000 rpm; and the upper layer was transferred into the same flask. Anhydrous sodium sulfate was subsequently added to the flask in which the upper layer had been collected, followed by mixing and filtration.

The filtrate was applied to a purification column [DSC-NH_2_ (2 g), Mega Bond Elut PSA (2 g), or DSC-Si (5 g)], which had been activated with ethyl acetate, and the eluate was collected in a flask. Further, 40 mL of ethyl acetate was added, and the eluate was collected in the same flask. The eluate was concentrated, at reduced pressure; dissolved in 5 mL of acetone; and concentrated again, at reduced pressure. When the purification column was not employed, the filtrate was concentrated, dissolved in 5 mL of acetone, and concentrated again, at reduced pressure. Acetone was added to the concentrate to obtain exactly 2 mL.

For linearity evaluation and matrix effect verification, the standard solutions, corresponding to 10%, 20%, 50%, 75%, 100%, 125%, and 150% of the analytical concentration, were added. Additionally, while validating, 0.1% l-gulonic acid γ-lactone was added.

## Results and discussion

### Development of the pretreatment method

For the extraction method (referring to USP), 20 mL of acetone was utilized for 2 g of DKT [8]. First, the recovery rates of each pretreatment step were obtained, employing only the pesticide standard solution, to investigate the interaction of the pesticides with each preparation step. Afterward, a recovery test, utilizing DKT, was performed to investigate the influence of the matrix derived from DKT. The evaluation criteria to validate the method were 70–120%, for recovery test employing DKT. In developing an analytical method, a recovery of 70–120% must be achieved with a combination of multiple treatments. Therefore, the acceptance criterion for the recovery rate in each process without DKT was set at ≥ 80%.

### Evaluation of the C18 SPE column purification process with the standard solution

The C18 column purification process is generally employed to purify pesticides from extracts [13]. To efficiently purify the extract, a SepPak C18 (5 g) column with a large amount of silica gel support was selected. Acetonitrile, generally employed for pesticide residue analysis in food was utilized for the elution. An additional elution was performed with acetone, which was used in the extraction process. To confirm the elution pattern of the pesticides in the C18 column purification step, the pesticide standard solution was added to the C18 column, and the recovery rates of each pesticide with acetonitrile and acetone were determined. The recovery rate was the average of the results of the two tests.

The recovery rate is ≥ 80% for 90 of 91 tested compounds (data not shown). The recovery rates of bromopropylate with acetonitrile and acetone are 47.6% and 27.0%, respectively. Bromopropylate exhibits a high affinity for the C18 column, and it has been demonstrated that the recovery rate may be unstable in the recovery test with DKT.

#### Evaluation of the liquid–liquid distribution and the dehydration process with the standard solution

The liquid–liquid distribution, from aqueous phase to organic phase, and the dehydration steps were demonstrated for the purpose of utilizing the normal-phase column process in the next step. The recovery test, utilizing the pesticide standard solution, for the liquid–liquid distribution and dehydration was conducted [14]. The recovery rate was the average of the results of the three tests.

The recovery rate is ≥ 80% for 86 of the 91 tested compounds (data not shown). The compounds whose recovery rates in the first distribution operation were ˂ 80% were fensulfothion (72.1%), fensulfothion oxon (60.2%), fensulfothion-oxon-sulfone (78.2%), fenthion-oxon-sulfoxide (66.4%), and fenthion sulfoxide (78.1%). Each of the compounds was not recovered in the second and third operations.

The recovery rates of fensulfothion, fensulfothion oxon, fensulfothion-oxon-sulfone, fenthion-oxon-sulfoxide, and fenthion sulfoxide did not increase even after repeated partitioning operations. It was speculated that the efficiency of the liquid–liquid distribution was not responsible for the decrease in the recovery rate.

#### Evaluation of the normal-phase column purification process with the standard solution

DSC-NH_2_ (2 g), Mega Bond Elut PSA (2 g), and DSC-Si (5 g) were selected as the normal-phase columns to retain highly polar substances [15], and ethyl acetate was selected as the eluting solvent [15]. To confirm the elution pattern of the pesticide in the normal-phase column purification process, the standard solution was added to the normal-phase column, and the recovery rate of each compound with ethyl acetate was determined. The recovery rate is the average of the results of the three tests.

The recovery rates with DSC-NH_2_ (2 g) are ˃ 80% for all the compounds (data not shown). The compounds whose rates were ˂ 80% in the Bond Elut PSA (2 g) column are fenchlorphos oxon (79.5%) and delta-BHC (78.2%) and those whose rates were ˂ 80% in DSC-Si (5 g) were *N*-desethyl-pirimiphos-methyl (0.3%), fenthion-oxon (74.7%), fensulfothion oxon (0%), and fenthion-oxon-sulfoxide (14.1%). In the study with the standard solution, the method of elution, employing 2 g of DSC-NH_2_ with ethyl acetate, was the most appropriate.

#### Evaluation of the pretreatment methods

The recovery test in DKT was conducted in the case of using no purification column, DSC-NH_2_ (2 g), Mega Bond Elut PSA (2 g), and DSC-Si (5 g). These evaluations included the C18 column purification and the liquid–liquid distribution processes, as shown in Fig. 1. The recovery rate was the average of the results of the three tests (Table 2).

**Table 2:** Evaluation of the purification columns

Purification column process	Events outside the evaluation range		Compounds outside the evaluation range^a^
No column	Recovery rate	< 70%	Fenthion oxon (63.5), heptachlor (56.3), pirimiphos-methyl (66.8), and profenophos(44.7)
		> 120%	*cis*-Chlorfenvinphos (136.6), fensulfothion oxon (132.5), and fenthion-oxon-sulfoxide (122.2)
	RSD	> 20%	–
Mega Bond Elut PSA (2 g)	Recovery rate^b^	< 70%	Delta-BHC (65.9), fenchlorphos oxon (38.2), paraoxon-methyl (45.8), phosmet (40.5), and profenophos (19.2)
		> 120%	Fensulfothion (126.0) and fensulfothion oxon (142.2)
	RSD	> 20%	–
DSC-NH_2_ (2 g)	Recovery rate^b^	< 70%	Cyhalothrin (67.8), deltamethrin (65.9), delta-BHC (63.2),and profenophos (26.9)
		> 120%	Dicofol (122.1)
	RSD	> 20%	–
DSC-Si (5 g)	Recovery rate^b^	< 70%	Fensulfothion (39.3), fensulfothion oxon (0), fenthion oxon(55.1), fenthion-oxon-sulfoxide (3.0), fenthion sulfoxide (26.5), heptachlor (56.7), *N*-desethyl-pirimiphos methyl (4.6), and pirimiphos-methyl (67.5)
		> 120%	*cis*-Chlorfenvinphos (134.4), dicofol (154.9), endrin (121.5), fenthion-oxon-sulfone (124.8), flucythrinate (122.0), and fluvalinate(124.4)
	RSD	> 20%	Fensulfothion (26.7), fenthion-oxon-sulfoxide (21.5), fenthion sulfoxide (39.4), *N*-desethyl-pirimiphos-methyl (81.4)

^a^Number of bracket were recovery rate (%)

^b^For the underlined compounds, the recovery rates without DKT were ˂ 80%

DSC-NH_2_ (2 g) was mostly pesticides with recovery rates of 70–120% (Table 2). The recovery rates of cyhalothrin, deltamethrin, delta-BHC, and profenophos were ˂ 70% with DKT (Table 2) but were ˃ 80% without DKT. It was speculated that the DKT-derived matrices affected the pretreatment and GC–MS/MS analysis processes. The combination of the SepPak C18 (5 g) column and DSC-NH_2_ (2 g) column was the most excellent pretreatment method for the removal of the interfering components derived from DKT to ensure the recovery of the pesticide to be analyzed. DSC-NH_2_ and Mega Bond Elut PSA are silica-based sorbents modified with an amino propyl group and an ethylenediamine-*N*-propyl group, respectively. Both sorbents exhibit a weak nonpolar interaction in nonpolar solvents. However, their different structure may cause a difference in the ability to remove the matrices in the DKT. In addition, since DSC-Si has a strong polar interaction even in nonpolar solvents, highly polar pesticides are strongly retained, and the recovery rate is low.

### Validation

In the GC analysis, a calculated value may be lower or higher than the true value due to the matrix effect [16]. Stable isotopes, matrix calibration curve, or matrix effect inhibitors is a method of solving the challenge of matrix effect [16–18]. However, it is challenging to prepare the stable isotopes of 107 compounds. Moreover, when preparing the matrix calibration curve, the representative samples that generally exhibit constant matrix effects are required [17, 19]. Therefore, in this study, the matrix effect inhibitor (l-gulonic acid γ-lactone) was employed for the validation of the test method. [19] Additionally, to deepen the discussion, the matrix effect of each pesticide was evaluated.

The validation was conducted based on the evaluation criteria, as shown in Table 3, [8, 17, 20]. For the evaluation of the linearity, standard solutions corresponding to 10%, 20%, 50%, 75%, 100%, 125%, and 150% of the maximum residue limits (MRLs) were added to acetone and the sample solution, respectively. From each analysis, a deviation in the back-calculated concentration, from the true concentration, was calculated (Table 4). Further, for the evaluation of the true value and specificity, the standard solutions corresponding to 10%, 20%, 50%, 100%, and 150% of the MRLs were added to each sample, respectively. The recovery and relative standard deviation (RSD) (*n* = 3, for each concentration) were also calculated (Table 4). They were calculated only when the ion ratio and retention time satisfied the criteria, as shown in Table 3. For the evaluation of the matrix effect, the standard solutions corresponding to 10%, 20%, 50%, 75%, 100%, 125%, and 150% of MRLs were added to acetone and the sample solution, respectively. The recovery rate of the sample solution to acetone was calculated at each concentration, and the deviation, from 100%, was assumed to be the matrix effect (Table 5).

**Table 3:** Evaluation criteria for validation

Validation parameters	Validation criteria
Linearity	≤ ± 20%^a^
Recovery	70–120% (*n* = 3)
Precision	≤ 20% (*n* = 3)
Ion ratio	± 30%^b^
Retention time	< ± 0.1 min^c^
LOQ	≤ MRL^d^

^a^Deviation of back-calculated concentration from the true concentration

^b^Ion ratio of the samples should be within ± 30% of the standards

^c^Retention time of the samples should be within ± 0.1 min of the standards

^d^Lowest concentration (mg/kg) satisfying the other criteria

**Table 4:** Validation results

Standard reagents name	Linearity													
	Standard solutions (acetone)							DKT standard solutions (matrix matched)						
	10%	20%	50%	75%	100%	125%	150%	10%	20%	50%	75%	100%	125%	150%
Alachlor	− 1.4	− 4.3	− 1.0	− 1.8	6.9	− 1.7	− 1.3	− 2.6	− 2.0	1.1	2.0	1.3	− 4.0	1.6
Aldrin	− 14.0	− 0.6	− 1.2	2.4	2.5	1.7	− 2.7	− 6.3	− 6.3	3.0	− 1.5	3.4	0.9	− 1.9
Dieldrin	− 9.1	− 4.1	0.5	0.0	5.1	− 0.5	− 1.9	− 8.8	− 2.6	1.7	2.1	1.3	− 1.7	0.0
Azinphos-ethyl	70.9	4.1	− 4.9	− 8.9	5.2	− 3.6	2.6	26.0	5.8	− 2.7	− 2.9	− 0.5	− 2.8	3.0
AZINPHOS-methyl	93.9	8.5	− 6.6	− 10.0	4.1	− 1.4	2.0	27.8	11.2	− 4.1	− 4.3	0.1	− 2.7	3.1
Bromophos-ethyl	7.6	− 0.4	− 1.2	− 4.2	3.8	0.6	− 0.9	3.3	− 2.6	2.2	0.5	− 1.0	− 2.2	1.6
Bromophos-methyl	3.1	− 2.2	− 0.7	− 2.1	4.3	− 1.5	− 0.2	2.6	− 1.5	0.6	0.6	0.1	− 2.3	1.3
Bromopropylate	− 12.1	− 6.9	1.1	− 0.1	7.6	− 1.7	− 2.1	− 12.2	− 4.1	2.9	2.0	2.1	− 1.2	− 0.8
*cis*-Chlordane	1.3	− 4.0	− 1.4	− 1.8	6.4	− 1.8	− 0.9	5.8	3.9	− 0.2	− 0.9	− 1.9	− 0.7	1.5
*trans*-Chlordane	− 1.0	− 1.7	− 0.7	− 2.9	6.2	− 1.8	− 0.7	4.4	− 0.3	0.4	− 0.1	− 0.1	− 2.0	1.4
Oxychlordane	− 12.5	− 6.1	5.4	− 1.3	4.1	− 0.3	− 1.7	14.2	− 2.7	1.9	− 0.1	− 1.6	− 3.8	3.2
*cis*-Chlorfenvinphos	2.1	− 3.6	− 0.9	− 2.2	5.6	− 1.7	− 0.6	− 2.4	− 2.3	0.9	0.3	1.8	− 1.8	0.3
*trans*-Chlorfenvinphos	3.6	− 3.0	− 1.6	− 2.8	6.6	− 2.2	− 0.5	− 1.8	− 2.6	0.7	0.7	1.6	− 2.0	0.5
Chlorpyriphos-ethyl	5.1	− 2.1	− 1.0	− 1.7	3.2	− 1.0	− 0.2	4.6	− 0.9	0.2	0.2	− 0.1	− 1.7	1.2
Chlorpyriphos-methyl	− 4.5	− 3.6	0.5	− 1.1	5.0	− 1.6	− 0.8	1.9	− 2.4	0.8	0.6	0.4	− 1.9	0.9
Chlorthal-dimethyl	− 5.6	− 7.1	− 2.5	2.8	6.3	− 2.1	− 1.6	− 0.7	− 0.6	− 1.0	0.9	1.3	− 1.3	0.2
Cyfluthrin	45.3	− 0.3	− 5.4	− 7.8	9.3	− 4.5	1.4	18.8	1.2	− 1.9	− 2.0	1.1	− 2.8	2.1
Cyhalothrin	24.0	− 4.7	− 3.9	− 4.2	7.9	− 2.7	− 0.2	6.8	− 1.8	− 1.0	− 0.3	1.9	− 2.1	0.8
Cypermethrin	43.5	− 2.2	− 5.2	− 6.5	8.4	− 3.3	0.7	14.3	0.2	− 1.6	− 1.2	1.0	− 2.3	1.6
*o,p*'-DDE	− 7.6	− 4.4	1.4	− 0.4	4.4	− 0.9	− 1.3	− 7.6	− 3.0	2.5	2.0	0.2	− 1.4	0.2
*p,p*'-DDE	− 9.1	− 4.5	1.4	0.0	4.5	− 0.8	− 1.5	− 8.5	− 3.2	2.5	2.1	0.6	− 1.3	0.0
*o,p*'-DDD	− 7.9	− 4.7	0.8	− 0.6	5.6	− 0.9	− 1.7	− 8.5	− 2.8	1.7	1.5	1.8	− 1.5	− 0.2
*p,p*'-DDD	4.7	− 2.6	− 0.1	− 2.4	4.4	− 2.3	0.3	− 0.7	− 1.4	0.8	1.5	− 0.1	− 2.4	1.2
*o,p*'-DDT	− 5.1	− 5.0	0.2	− 1.1	6.1	− 1.4	− 1.4	− 8.0	− 3.3	2.0	1.4	1.6	− 1.1	− 0.4
*p,p*'-DDT	14.2	− 1.6	− 0.7	− 4.2	4.8	− 3.1	1.1	5.2	0.1	0.1	0.7	− 0.6	− 2.6	1.9
Deltamethrin	88.2	− 2.3	− 6.6	− 8.3	9.4	− 4.0	1.2	22.7	1.2	− 1.9	− 2.0	0.6	− 2.4	2.0
Diazinon	− 1.4	− 3.8	− 0.5	− 1.5	5.7	− 1.7	− 0.9	− 4.3	− 3.1	1.3	1.5	1.4	− 2.2	0.5
Dicofol	− 9.9	− 3.0	− 4.4	1.1	6.7	1.2	− 3.6	− 7.0	− 1.4	7.7	− 0.2	− 4.3	− 0.4	1.4
α-Endosulfan	− 9.6	− 4.5	1.6	− 0.1	5.0	− 1.2	− 1.4	− 8.3	− 2.7	2.1	1.6	1.5	− 1.9	0.1
β-Endosulfan	− 6.3	− 4.9	0.4	− 1.2	6.4	− 1.3	− 1.6	− 8.6	− 2.0	1.4	1.6	1.9	− 1.8	− 0.1
Endosulfan-sulfate	− 7.0	− 5.7	0.4	− 1.4	7.4	− 1.6	− 1.7	− 6.7	− 3.5	1.2	1.3	2.6	− 2.0	− 0.1
Endrin	− 3.2	7.7	− 4.4	− 0.8	2.7	− 0.8	− 0.1	− 5.5	− 1.3	− 2.5	2.9	2.0	− 0.4	− 1.0
Ethion	− 8.0	− 6.9	0.6	− 0.5	7.1	− 1.8	− 1.7	− 10.4	− 4.5	2.1	2.0	2.6	− 1.6	− 0.6
Etrimphos	− 7.5	− 4.3	1.6	− 1.5	5.0	− 0.4	− 1.7	− 8.4	− 2.3	2.5	1.1	1.1	− 1.4	0.0
Fenchlorphos	− 3.9	− 3.6	1.0	− 0.9	3.8	− 1.0	− 0.8	− 3.2	− 2.2	1.8	1.5	− 0.2	− 1.6	0.7
Fenchlorphos oxon	1.4	− 3.4	0.5	− 2.2	4.6	− 1.9	− 0.2	36.0	9.6	− 4.9	− 3.2	− 7.1	8.0	− 1.3
Fenitrothion	8.0	− 1.9	− 1.4	− 3.1	5.3	− 2.4	0.2	6.7	− 0.8	− 0.7	− 0.3	1.0	− 1.9	1.0
Fenpropathrin	5.0	− 3.7	− 0.6	− 3.4	6.0	− 1.7	− 0.5	− 4.0	− 1.3	1.6	− 0.4	1.8	− 1.6	0.3
Fensulfothion	37.7	− 2.9	− 4.6	− 6.8	9.7	− 5.0	1.3	16.8	− 0.2	− 0.9	− 1.8	0.7	− 2.6	2.0
Fensulfothion oxon	39.1	− 0.4	− 0.1	− 8.0	5.6	− 5.9	3.5	8.6	3.5	− 0.3	− 2.5	1.8	− 4.4	2.9
Fensulfothion-oxon-sulfone	− 10.0	− 1.2	3.1	− 1.9	4.6	− 3.4	0.5	− 20.6	4.4	1.4	4.4	− 0.2	− 2.8	0.8
Fensulfothion-sulfone	18.2	− 6.6	− 4.7	− 2.8	9.5	− 4.7	0.3	− 4.7	0.1	− 1.1	0.8	4.1	− 4.2	1.0
Fenthion	0.3	− 2.3	− 1.2	− 1.6	4.8	− 1.2	− 0.8	2.9	− 1.3	0.3	0.8	− 0.1	− 2.0	1.2
Fenthion oxon	− 0.9	− 2.9	0.9	− 2.0	5.1	− 3.2	0.4	0.9	− 6.0	2.1	0.1	2.3	− 2.7	0.7
Fenthion-oxon-sulfone	21.9	3.3	0.1	− 9.5	4.8	− 0.5	0.5	6.3	14.1	− 3.3	− 6.8	2.7	− 0.8	1.2
Fenthion-oxon-sulfoxide	38.0	10.6	− 3.1	− 14.4	9.1	− 2.9	1.6	− 7.7	11.7	1.1	− 4.7	1.9	− 2.7	1.9
Fenthion-sulfone	9.7	1.1	− 5.0	− 4.3	8.4	− 2.5	− 0.4	6.3	3.9	0.5	− 3.9	1.3	− 1.9	1.6
Fenthion sulfoxide	62.2	− 0.2	− 6.5	− 6.3	7.0	− 4.1	1.8	19.1	4.7	− 1.1	− 5.2	2.6	− 4.5	3.2
Fenvalerate	35.6	− 3.7	− 4.7	− 5.8	8.6	− 2.9	0.1	9.9	− 1.3	− 1.3	− 1.0	1.9	− 2.0	0.9
Flucythrinate	31.5	− 1.1	− 4.7	− 7.3	10.3	− 4.4	0.7	13.2	1.4	− 1.6	− 2.3	1.9	− 2.3	1.4
Fluvalinate	46.6	− 1.7	− 4.6	− 8.3	10.6	− 5.8	1.8	26.3	3.5	− 2.8	− 2.5	0.4	− 2.6	2.4
Fonophos	− 1.4	− 2.2	− 0.6	− 1.1	4.5	− 1.5	− 0.5	− 1.4	− 0.3	0.8	0.2	0.8	− 2.1	1.0
Heptachlor	− 12.7	− 8.4	3.7	1.5	5.4	− 1.9	− 1.7	− 13.0	− 4.9	3.2	3.5	1.7	− 2.2	− 0.3
*cis*-Heptachlorepoxide	0.7	− 4.1	− 1.8	− 1.0	6.0	− 1.9	− 0.9	5.9	− 1.6	− 0.1	− 0.4	1.8	− 3.0	1.4
*trans*-Heptachlorepoxide	− 4.3	− 0.7	− 5.5	2.9	5.4	− 2.0	− 1.1	− 6.8	16.4	− 1.2	0.5	− 4.8	− 2.8	3.8
Hexachlorobenzene	− 1.1	− 1.8	0.7	− 0.6	1.9	− 1.0	− 0.1	1.7	1.5	1.0	1.0	− 1.9	− 2.4	2.1
α-Hexachlorocyclohexane	− 1.2	− 2.7	0.3	− 1.3	3.8	− 1.4	− 0.4	− 1.3	− 0.7	1.3	0.6	0.2	− 2.2	1.2
β-hexachlorocyclohexane	− 2.8	− 3.2	− 0.8	− 1.7	6.2	− 1.6	− 1.1	− 1.6	− 2.0	0.4	0.5	1.6	− 1.7	0.3
δ-Hexachlorocyclohexane	1.2	− 3.3	0.7	− 2.2	4.2	− 1.6	− 0.2	− 0.2	− 1.2	1.0	0.1	0.6	− 1.7	0.8
ε-Hexachlorocyclohexane	− 13.7	− 7.5	4.5	− 0.8	6.8	− 1.4	− 2.1	− 15.2	− 4.5	4.0	3.0	2.5	− 2.6	− 0.3
γ-Hexachlorocyclohexane	− 3.7	− 3.4	0.4	− 1.1	4.6	− 1.3	− 0.8	− 4.2	− 2.1	1.7	1.0	0.9	− 2.2	0.7
Malaoxon	4.5	− 2.8	− 0.4	− 3.5	6.7	− 3.3	0.3	4.4	− 0.5	− 0.5	− 0.1	0.1	− 0.6	0.5
Malathion	− 4.5	− 4.5	0.2	− 1.6	6.6	− 2.0	− 1.1	− 5.4	− 3.0	1.2	0.9	2.3	− 2.0	0.1
Mecarbam	5.6	− 1.1	− 5.0	− 0.5	5.6	− 1.6	− 0.7	6.0	2.7	− 0.9	− 3.6	3.3	− 2.3	1.1
Methacriphos	− 0.4	− 2.8	0.0	− 2.1	4.9	− 1.7	− 0.5	− 1.9	0.0	0.9	0.6	1.0	− 3.3	1.6
Methidathion	10.1	− 1.6	− 1.6	− 3.7	5.5	− 2.4	0.3	0.7	− 1.3	0.5	− 0.3	1.5	− 1.8	0.6
Methoxychlor	26.1	1.6	− 2.3	− 6.6	6.5	− 4.7	2.1	10.8	1.7	− 1.0	0.1	− 0.7	− 3.2	2.5
Mirex	− 6.1	46.6	− 22.9	− 11.9	1.3	− 0.8	5.2	− 6.6	48.0	− 22.8	− 10.5	− 2.6	0.4	5.8
Parathion-ethyl	21.2	− 1.1	− 3.3	− 4.9	6.0	− 1.8	0.1	20.3	− 1.1	− 1.6	− 1.3	0.5	− 1.8	1.5
Paraoxon-ethyl	15.1	− 1.0	− 3.0	− 3.6	5.5	− 2.0	0.1	18.5	1.7	− 2.7	− 1.1	0.8	− 2.2	1.7
Parathion-methyl	7.8	− 2.0	− 2.0	− 2.7	5.1	− 1.7	− 0.2	9.1	0.1	− 1.0	− 0.3	0.1	− 1.7	1.2
Paraoxon-methyl	15.5	0.5	− 1.6	− 5.1	4.1	− 1.0	0.3	62.4	5.4	− 2.4	− 8.2	− 2.2	4.8	− 0.3
Pentachloroanisole	0.0	− 2.0	1.1	− 1.2	1.9	− 1.0	0.0	3.2	1.7	1.2	0.8	− 2.6	− 2.2	2.3
*cis*-Permethrin	20.5	0.1	0.2	− 9.7	7.2	− 1.5	0.1	− 2.1	− 4.7	1.2	1.4	1.2	− 1.4	0.0
*trans*-Permethrin	8.7	− 6.0	− 3.0	− 1.8	6.1	− 0.4	− 1.6	− 4.3	− 2.2	0.7	0.4	2.9	− 2.4	0.3
Phosalone	26.7	− 1.7	− 3.5	− 5.8	7.5	− 3.4	0.8	11.5	0.7	− 0.7	− 1.6	0.9	− 2.3	1.6
Phosmet	24.8	− 1.0	− 3.2	− 6.1	7.2	− 3.5	1.0	9.2	2.1	− 1.5	− 1.9	0.7	− 0.5	0.6
Pirimiphos-ethyl	− 3.2	− 1.3	− 2.0	− 2.9	7.8	− 1.5	− 1.4	− 0.8	− 2.0	− 0.2	0.6	2.2	− 2.3	0.5
Pirimiphos-methyl	− 19.1	− 7.2	4.1	2.1	6.1	− 1.2	− 2.7	− 18.6	− 6.3	4.5	4.1	3.1	− 1.8	− 1.5
*N*-Desethyl-pirimiphos-methyl	− 12.6	− 5.5	1.7	0.0	6.7	− 1.3	− 2.1	− 11.7	− 2.4	2.2	1.8	2.2	− 1.7	− 0.4
Procymidone	− 0.5	− 2.9	− 0.7	− 2.3	6.1	− 1.9	− 0.7	− 3.6	− 3.7	1.8	0.9	1.6	− 2.1	0.4
Profenophos	6.8	− 2.0	− 1.8	− 2.7	5.1	− 1.7	− 0.2	− 13.4	− 13.3	1.8	5.2	5.4	− 1.6	− 2.5
Prothiophos	− 8.1	− 3.8	1.0	− 0.6	5.2	− 1.3	− 1.3	− 8.6	− 2.5	2.7	1.6	0.6	− 1.5	0.1
Quinalphos	4.3	− 2.5	− 1.6	− 2.8	7.2	− 3.7	0.3	1.9	− 2.2	0.0	0.2	2.3	− 3.0	1.1
Quintozene	− 0.4	− 2.7	0.5	− 1.5	3.2	− 0.8	− 0.5	0.0	− 0.7	1.7	0.6	− 0.7	− 2.0	1.4
Pentachloroaniline	− 3.3	− 2.6	1.6	− 1.7	3.3	− 0.8	− 0.6	− 4.5	− 2.1	2.0	1.5	− 0.2	− 1.7	0.7
Methyl pentachlorophenyl Sulfide	− 0.9	− 2.1	1.0	− 0.6	1.5	− 0.7	− 0.1	− 2.6	0.1	2.2	1.3	− 2.0	− 1.5	1.4
s-421	a	25.2	− 1.7	− 1.3	− 2.2	− 8.2	6.7	a	− 14.5	16.5	− 2.1	− 5.6	− 1.7	2.6
Tecnazene	4.2	− 1.5	0.4	− 2.5	2.4	− 0.6	− 0.1	3.2	2.9	0.3	0.4	− 1.5	− 2.6	2.3
Tetradifon	1.9	− 3.4	− 0.8	− 2.3	5.4	− 1.3	− 0.8	− 0.4	− 2.1	1.0	0.5	0.9	− 2.0	0.8
Vinclozolin	− 2.3	− 3.1	0.9	− 2.6	5.4	− 1.7	− 0.6	− 3.1	− 1.9	1.2	0.8	0.8	− 1.5	0.4

Standard reagents name	Recovery rate (%)					RSD (%)					LOQ
	10%	20%	50%	100%	150%	10%	20%	50%	100%	150%	
Alachlor	A	111.9	99.6	91.0	95.6	a	7.6	2.8	3.9	1.8	0.01
Aldrin	91.2	85.9	89.0	87.2	90.6	4.7	7.5	8.5	2.0	1.4	0.005
Dieldrin	91.5	90.7	90.3	87.4	89.9	1.8	2.3	1.5	0.8	1.8	0.005
Azinphos-ethyl	124.9	125.5	120.4	111.8	112.5	0.8	5.7	1.0	1.8	3.1	0.05
AZINPHOS-methyl	116.4	116.5	110.6	101.0	102.1	1.1	5.7	3.3	1.3	4.8	0.2
Bromophos-ethyl	99.7	101.5	99.2	94.5	98.3	3.0	3.5	2.5	2.8	0.4	0.005
Bromophos-methyl	93.0	94.2	93.8	91.6	94.8	1.2	2.0	1.5	2.3	0.6	0.005
Bromopropylate	101.1	98.0	94.4	89.3	92.7	0.9	0.9	0.6	2.2	0.8	0.3
*cis*-Chlordane	91.2	91.7	88.6	84.7	89.9	1.1	4.7	2.8	2.3	3.2	0.005
*trans*-Chlordane	94.4	94.2	93.8	87.7	92.5	4.2	0.9	1.2	1.5	1.5	0.005
Oxychlordane	100.1	91.5	91.6	84.0	91.1	8.7	13.6	4.7	4.0	7.2	0.005
*cis*-Chlorfenvinphos	97.5	97.5	95.9	91.2	94.0	0.4	1.1	0.2	1.6	0.3	0.05
*trans*-Chlorfenvinphos	99.5	98.5	96.9	91.5	94.6	0.3	1.1	0.8	1.8	0.4	0.05
Chlorpyriphos-ethyl	95.8	95.5	95.6	91.3	94.0	3.1	0.7	1.2	1.1	0.8	0.02
Chlorpyriphos-methyl	92.0	92.1	91.7	88.3	92.4	1.0	1.8	1.6	1.7	0.6	0.01
Chlorthal-dimethyl	90.1	91.9	91.9	90.8	92.6	5.4	2.1	0.2	2.8	2.0	0.001
Cyfluthrin	125.3	123.2	119.9	109.6	113.2	2.4	4.9	1.8	2.5	1.8	0.05
Cyhalothrin	111.5	108.8	102.8	95.0	97.6	0.2	2.4	0.2	2.4	1.1	0.2
Cypermethrin	119.3	119.0	112.1	101.6	104.6	0.7	2.8	0.8	2.5	1.6	0.2
*o,p*'-DDE	91.9	91.4	90.6	86.7	89.9	0.9	1.4	0.8	1.0	1.4	0.1
*p,p*'-DDE	92.9	92.0	91.2	87.6	91.0	0.7	1.2	0.8	0.4	1.0	0.1
*o,p*'-DDD	93.1	92.3	90.9	87.0	90.6	1.2	0.3	0.6	0.9	0.2	0.1
*p,p*'-DDD	96.9	95.7	92.8	88.8	91.3	0.7	2.0	1.3	1.3	1.5	0.1
*o,p*'-DDT	95.9	94.4	92.0	87.0	89.8	0.9	1.7	0.9	0.6	1.0	0.1
*p,p*'-DDT	103.1	100.8	96.7	91.2	93.3	0.9	2.2	0.8	1.8	2.4	0.1
Deltamethrin	133.6	134.2	124.3	110.3	112.7	3.5	6.1	1.3	3.5	2.5	0.5
Diazinon	95.4	94.9	94.0	90.3	94.0	0.8	0.8	0.8	1.9	1.0	0.05
Dicofol	110.1	107.8	112.8	93.6	105.1	22.2	12.0	14.9	14.5	9.5	0.1
α-Endosulfan	90.0	91.1	89.5	86.8	89.7	0.9	2.1	1.0	0.6	1.0	0.3
β-Endosulfan	95.0	93.8	90.0	85.4	88.6	0.5	1.7	0.6	0.5	1.4	0.3
Endosulfan-sulfate	95.3	93.9	91.9	87.7	90.9	0.6	0.4	0.4	1.6	1.7	0.3
Endrin	90.5	90.0	90.9	89.9	88.3	9.7	6.7	2.4	2.8	3.3	0.005
Ethion	100.7	97.3	93.2	88.3	90.8	1.4	1.5	0.4	1.5	1.2	0.2
Etrimphos	93.3	92.4	92.3	87.4	91.1	1.5	2.8	0.9	0.8	0.6	0.005
Fenchlorphos	95.4	92.8	93.1	89.9	93.0	1.3	1.2	0.5	1.3	0.6	0.01
Fenchlorphos oxon	44.8	47.5	45.5	43.6	46.2	12.8	14.8	7.9	5.1	2.6	C
Fenitrothion	95.1	94.7	94.4	90.8	93.9	0.8	1.0	0.8	1.8	0.1	0.05
Fenpropathrin	112.4	108.9	104.2	97.9	99.4	2.9	2.6	0.6	1.7	1.3	0.003
Fensulfothion	120.1	128.0	123.2	113.2	116.1	7.8	3.2	4.2	5.7	0.4	0.05
Fensulfothion oxon	122.7	125.2	117.9	110.5	112.3	8.0	8.7	3.1	2.7	0.4	0.025
Fensulfothion-oxon-sulfone	113.8	107.7	101.1	97.4	97.8	6.8	8.7	2.8	3.2	4.3	0.01
Fensulfothion-sulfone	106.0	115.1	110.2	99.6	102.5	8.0	3.7	1.1	1.2	1.6	0.005
Fenthion	87.8	91.0	90.1	87.5	91.1	4.0	2.0	1.7	3.5	2.5	0.005
Fenthion oxon	89.9	89.6	91.7	88.9	92.2	0.8	4.0	1.9	1.4	2.9	0.005
Fenthion-oxon-sulfone	81.7	93.8	83.9	84.9	83.7	16.4	12.4	4.8	5.9	5.2	0.01
Fenthion-oxon-sulfoxide	b	123.6	113.6	100.7	101.8	b	9.5	4.8	2.6	3.5	0.025
Fenthion-sulfone	84.7	82.7	84.4	74.8	78.8	6.6	5.3	3.0	0.2	3.1	0.005
Fenthion sulfoxide	144.7	141.4	124.5	115.5	113.9	5.6	6.5	4.6	4.5	3.5	0.05
Fenvalerate	123.0	122.0	110.4	99.9	102.3	1.0	2.4	1.3	2.7	2.2	0.75
Flucythrinate	129.3	131.1	127.1	114.2	119.4	2.1	4.2	0.6	1.9	1.5	0.05
Fluvalinate	125.7	127.1	121.6	111.2	115.4	1.4	4.9	1.1	3.4	2.5	0.05
Fonophos	90.6	90.1	90.9	87.5	91.8	2.2	1.1	1.1	0.3	1.0	0.005
Heptachlor	90.3	91.6	90.8	88.1	91.7	5.2	0.7	3.0	1.0	1.0	0.005
*cis*-Heptachloroepoxide	92.3	90.7	90.6	87.7	92.0	2.3	3.6	2.4	0.9	2.5	0.005
*trans*-Heptachloroepoxide	b	102.6	95.1	88.1	96.8	b	11.4	3.6	4.3	0.8	0.01
Hexachlorobenzene	81.9	81.3	80.7	78.6	83.2	1.8	2.3	1.8	0.3	0.6	0.01
α-Hexachlorocyclohexane	88.1	87.3	87.4	84.8	88.4	1.7	1.2	1.9	1.5	0.4	0.03
β-Hexachlorocyclohexane	94.0	93.7	93.7	89.8	93.5	0.8	0.1	0.3	1.3	0.5	0.03
δ-Hexachlorocyclohexane	93.9	94.2	93.5	91.0	93.8	0.9	1.7	0.2	2.0	0.5	0.03
ε-Hexachlorocyclohexane	93.3	94.3	93.5	91.9	94.2	2.7	1.6	1.0	3.3	1.1	0.03
γ-Hexachlorocyclohexane	89.5	88.9	89.3	86.0	89.6	0.6	1.0	1.0	1.4	0.2	0.06
Malaoxon	84.5	85.6	84.6	83.2	84.3	3.5	1.6	1.2	6.2	0.2	0.1
Malathion	93.4	93.3	92.9	89.8	92.7	0.3	1.0	0.1	2.0	0.9	0.1
Mecarbam	111.1	101.7	104.7	96.7	98.4	4.5	6.1	0.5	1.7	2.4	0.005
Methacriphos	83.2	82.3	82.5	80.0	84.7	2.5	2.5	2.5	1.3	1.0	0.005
Methidathion	100.4	100.7	99.2	94.0	95.8	0.8	1.3	0.6	1.9	1.0	0.02
Methoxychlor	115.6	112.7	109.8	102.4	105.0	1.8	3.7	1.4	2.2	2.8	0.01
Mirex	93.5	95.5	93.8	90.6	93.0	1.0	1.2	2.4	1.7	1.0	0.01
Parathion-ethyl	96.0	94.8	94.7	89.3	92.9	1.7	0.0	1.2	1.4	1.0	0.1
Paraoxon-ethyl	92.4	92.8	90.6	88.0	91.2	2.4	2.9	0.7	3.0	0.7	0.05
Parathion-methyl	94.9	93.9	94.8	91.4	94.5	1.3	1.5	0.9	2.6	0.5	0.02
Paraoxon-methyl	50.8	52.0	50.9	51.5	53.0	14.6	12.8	3.3	4.6	1.4	c
Pentachloroanisole	85.3	83.8	82.7	80.3	84.5	3.5	3.0	1.1	2.0	0.7	0.001
*cis*-Permethrin	114.7	107.4	104.3	95.1	96.9	0.4	2.1	2.6	1.7	1.4	0.2
*trans*-Permethrin	114.1	108.9	103.4	94.5	96.4	0.5	4.8	2.8	0.6	2.0	0.1
Phosalone	112.0	111.8	109.0	101.6	103.8	0.7	2.9	0.9	3.2	2.0	0.02
Phosmet	101.2	105.1	103.7	97.9	98.0	2.0	4.9	1.1	4.2	3.2	0.01
Pirimiphos-ethyl	100.2	95.7	98.5	93.4	98.0	0.7	1.4	1.0	2.5	0.6	0.005
Pirimiphos-methyl	95.3	94.3	94.0	91.8	94.5	0.7	0.3	0.9	1.0	0.6	0.4
*N*-Desethyl-pirimiphos-methyl	95.5	96.0	96.6	94.6	97.7	1.1	1.9	0.7	2.8	1.3	0.4
Procymidone	97.4	94.2	95.2	89.4	92.6	1.5	1.0	0.6	1.4	0.5	0.01
Profenophos	70.5	70.1	71.0	68.4	68.3	1.8	4.0	2.7	3.6	4.1	c
Prothiophos	98.3	98.8	97.7	93.6	96.3	2.3	2.2	0.9	1.6	1.1	0.005
Quinalphos	103.1	105.9	104.4	99.0	102.8	0.8	2.0	0.3	2.7	1.5	0.005
Quintozene	86.2	86.0	85.8	82.8	87.0	0.5	1.5	1.7	1.2	0.4	0.1
Pentachloroaniline	93.9	92.4	92.4	89.7	93.2	1.4	1.7	0.5	1.1	0.6	0.1
Methyl pentachlorphenyl Sulfide	92.4	91.7	91.4	88.1	91.2	1.5	1.8	0.8	0.6	0.9	0.1
s-421	a	87.2	103.8	90.4	90.7	a	16.3	10.9	7.7	8.8	0.01
Tecnazene	83.3	82.2	80.9	78.1	83.5	3.0	4.1	2.4	1.1	0.9	0.005
Tetradifon	99.5	97.4	95.7	90.7	93.4	1.2	0.6	1.0	1.9	1.3	0.03
Vinclozolin	92.0	93.1	91.2	88.4	95.0	1.6	0.9	1.4	1.9	7.0	0.04

Linearity, recovery, accuracy, and LOQ were evaluated according to the validation criteria in Table 3. The out-of-standard results are underlined. The recovery rate is displayed only when the ion ratio and retention time satisfied the criteria

^a^MRM2: no signal

^b^ion ratio out of the criteria

^c^All concentration results did not satisfy the validation criteria

**Table 5:** Matrix effect

	10%	20%	50%	75%	100%	125%	150%	Standard reagents name	10%	20%	50%	75%	100%	125%	150%
Alachlor	10.0	3.8	− 3.4	− 3.3	− 12.7	− 10.4	− 5.9	Fenthion-sulfone	− 10.2	− 12.2	− 13.8	− 19.0	− 25.0	− 19.5	− 18.5
Aldrin	− 13.5	− 20.9	− 9.3	− 15.5	− 11.1	− 12.4	− 10.8	Fenthion sulfoxide	53.7	50.6	29.6	20.2	12.3	15.5	17.0
Dieldrin	− 7.7	− 7.5	− 8.5	− 7.8	− 13.0	− 10.9	− 8.2	Fenvalerate	24.0	21.6	10.2	9.4	− 3.2	3.5	2.9
Azinphos-ethyl	26.0	33.7	21.9	24.7	9.6	16.1	15.2	Flucythrinate	31.3	34.9	27.6	28.5	11.9	23.5	21.2
Azinphos-methyl	14.9	24.0	10.0	11.4	− 0.4	1.6	3.7	Fluvalinate	27.8	34.2	22.3	26.2	7.1	21.7	18.2
Bromophos-ethyl	2.3	− 1.7	0.6	1.3	− 8.3	− 6.7	− 1.7	Fonophos	− 11.6	− 8.8	− 8.5	− 8.4	− 12.7	− 10.1	− 8.2
Bromophos-methyl	− 8.3	− 6.4	− 5.5	− 4.0	− 10.3	− 7.3	− 5.1	Heptachlor	− 14.1	− 8.5	− 10.8	− 8.1	− 12.9	− 10.0	− 8.2
Bromopropylate	0.4	− 1.1	− 5.4	− 5.9	− 12.9	− 8.0	− 7.2	*cis*-Heptachlorepoxide	− 8.1	− 8.1	− 7.4	− 8.1	− 12.1	− 9.5	− 6.2
*cis*-Chlordane	− 9.2	− 3.6	− 8.7	− 8.6	− 16.3	− 8.2	− 7.1	*trans*-Heptachlorepoxide	2.4	15.8	− 1.0	− 8.6	− 15.9	− 8.0	− 2.9
*trans*-Chlordane	− 2.0	− 6.2	− 7.0	− 5.4	− 13.6	− 8.3	− 6.2	Hexachlorobenzene	− 19.2	− 17.0	− 18.4	− 17.0	− 21.2	− 19.3	− 16.3
Oxychlordane	− 10.0	− 16.1	− 13.4	− 7.1	− 12.2	− 9.8	− 1.3	α-Hexachlorcyclohexane	− 13.3	− 11.5	− 12.5	− 11.7	− 16.3	− 14.0	− 12.0
*cis*-Chlorfenvinphos	− 2.1	− 1.3	− 3.9	− 3.8	− 9.8	− 6.7	− 5.9	β-Hexachlorcyclohexane	− 6.3	− 6.3	− 6.3	− 5.3	− 11.4	− 7.5	− 6.1
*trans*-Chlorfenvinphos	− 0.2	− 0.5	− 2.3	− 1.8	− 10.0	− 5.6	− 5.0	δ-Hexachlorcyclohexane	− 6.3	− 4.2	− 6.7	− 4.9	− 10.4	− 7.3	− 6.3
Chlorpyriphos-ethyl	− 3.0	− 3.8	− 5.3	− 4.9	− 9.8	− 7.6	− 5.7	ε-Hexachlorcyclohexane	− 9.5	− 4.4	− 7.5	− 3.4	− 10.6	− 8.0	− 5.1
Chlorpyriphos-methyl	− 7.2	− 9.7	− 9.1	− 7.4	− 12.8	− 9.0	− 7.1	γ-Hexachlorcyclohexane	− 11.0	− 10.1	− 10.4	− 9.9	− 15.0	− 12.6	− 10.5
Chlorthal-dimethyl	− 15.5	− 7.5	− 7.9	− 10.1	− 12.2	− 6.8	− 5.6	Malathion	− 6.9	− 5.7	− 7.1	− 6.0	− 12.0	− 8.4	− 7.4
Cyfluthrin	27.4	30.1	22.4	23.5	6.6	16.8	15.2	Malaoxon	− 18.8	− 15.2	− 16.4	− 13.2	− 21.2	− 13.6	− 15.8
Cyhalothrin	11.8	11.8	3.1	2.5	− 7.6	− 2.1	− 2.0	Mecarbam	5.8	6.0	4.5	− 3.4	− 2.6	− 1.4	1.1
Cypermethrin	20.0	22.7	13.0	12.9	− 1.3	6.4	5.9	Methacriphos	− 19.0	− 15.5	− 17.2	− 15.7	− 21.0	− 19.2	− 16.3
*o,p*'-DDE	− 7.2	− 7.2	− 8.5	− 7.7	− 13.5	− 10.3	− 8.7	Methidathion	− 0.5	2.0	− 0.2	0.4	− 7.2	− 3.1	− 3.5
*p,p*'-DDE	− 6.4	− 6.8	− 8.0	− 7.3	− 12.6	− 9.7	− 8.0	Methoxychlor	17.1	17.4	11.5	16.6	0.8	9.4	7.8
*o,p*'-DDD	− 8.1	− 7.4	− 9.4	− 8.6	− 13.9	− 11.3	− 9.5	Mirex	− 6.5	− 5.6	− 7.0	− 5.9	− 11.1	− 6.6	− 7.3
*p,p*'-DDD	− 2.6	− 2.3	− 6.1	− 4.0	− 12.0	− 8.4	− 7.6	Parathion-ethyl	− 2.5	− 5.3	− 5.2	− 3.5	− 12.0	− 7.3	− 6.0
*o,p*'-DDT	− 3.5	− 3.8	− 7.1	− 7.1	− 13.6	− 9.7	− 9.2	Paraoxon-ethyl	− 9.2	− 8.4	− 10.1	− 8.0	− 14.3	− 10.4	− 8.8
*p,p*'-DDT	2.9	3.1	− 3.0	0.1	− 10.2	− 5.1	− 5.0	Parathion-methyl	− 5.6	− 4.5	− 5.4	− 4.0	− 10.8	− 6.2	− 5.0
Deltamethrin	38.5	43.3	26.2	25.2	6.5	16.9	15.3	Paraoxon-methyl	− 56.6	− 53.6	− 49.4	− 49.3	− 50.2	− 43.4	− 46.6
Diazinon	− 5.1	− 4.6	− 5.6	− 4.9	− 11.7	− 8.6	− 6.9	Pentachloroanisole	− 13.2	− 13.3	− 16.6	− 15.1	− 20.5	− 17.8	− 15.0
Dicofol	35.6	20.3	24.5	7.3	− 3.3	5.6	12.4	*cis*-Permethrin	14.8	9.0	3.3	12.2	− 6.8	− 1.9	− 2.5
α-Endosulfan	− 10.4	− 9.5	− 10.3	− 9.2	− 13.6	− 11.3	− 9.2	*trans*-Permethrin	14.7	14.5	3.8	0.1	− 6.1	− 5.7	− 2.3
β-Endosulfan	− 5.2	− 4.5	− 9.3	− 8.4	− 15.0	− 12.0	− 10.3	Phosalone	11.2	15.5	10.1	10.5	− 1.2	6.2	5.5
endosulfan-sulfate	− 5.3	− 5.7	− 8.4	− 7.1	− 13.8	− 10.2	− 8.4	Phosmet	− 0.6	9.7	4.7	6.8	− 4.3	4.9	1.2
Endrin	− 0.1	− 13.2	− 8.0	− 7.5	− 11.9	− 11.3	− 12.7	Pirimiphos-ethyl	0.2	− 4.2	− 2.5	− 1.0	− 9.4	− 5.3	− 2.7
Ethion	0.1	− 1.1	− 6.4	− 6.3	− 12.9	− 9.3	− 8.6	Pirimiphos-methyl	− 5.4	− 5.4	− 6.1	− 4.8	− 9.2	− 7.2	− 5.5
Etrimphos	− 6.7	− 6.0	− 8.6	− 7.4	− 13.3	− 11.0	− 8.6	*N*-Desethyl-pirimiphos-methyl	− 5.6	− 2.2	− 3.9	− 2.5	− 8.1	− 4.4	− 2.2
Fenchlorphos	− 6.1	− 6.0	− 7.0	− 5.6	− 11.4	− 8.4	− 6.5	Procymidone	− 2.7	− 4.9	− 4.5	− 4.3	− 11.5	− 8.0	− 7.0
Fenchlorphos oxon	− 61.4	− 56.3	− 58.1	− 54.8	− 58.9	− 48.7	− 53.6	Profenophos	− 28.1	− 32.0	− 27.0	− 25.5	− 31.5	− 32.1	− 33.9
Fenitrothion	− 4.4	− 4.3	− 5.8	− 3.8	− 10.6	− 6.4	− 6.0	Prothiophos	− 0.2	− 0.4	− 1.5	− 1.3	− 7.8	− 4.0	− 2.4
Fenpropathrin	11.3	12.2	5.0	4.4	− 3.3	0.2	0.7	Quinalphos	3.5	4.3	4.7	6.0	− 2.1	3.4	3.3
Fensulfothion	53.7	50.6	29.6	20.2	12.3	15.5	17.0	Quintozene	− 14.2	− 12.6	− 13.2	− 12.4	− 17.4	− 15.2	− 12.5
Fensulfothion oxon	30.5	37.5	17.6	22.0	9.7	14.9	11.8	Pentachloroaniline	− 7.1	− 6.4	− 7.1	− 4.6	− 10.9	− 8.7	− 6.6
Fensulfothion-oxon-sulfone	6.9	16.9	2.6	9.5	− 2.5	2.5	1.8	Methyl pentachlorphenyl sulfide	− 9.1	− 6.5	− 8.0	− 7.6	− 12.5	− 10.1	− 8.1
Fensulfothion-sulfone	− 3.3	18.3	9.3	8.1	− 1.4	3.9	3.9	s-421	(no data)	− 23.0	13.0	− 8.5	− 12.4	− 3.7	− 14.1
Fenthion	− 12.4	− 11.2	− 9.1	− 7.9	− 14.1	− 10.5	− 7.9	Tecnazene	− 17.9	− 14.0	− 18.1	− 15.6	− 21.2	− 19.8	− 16.2
Fenthion oxon	− 10.8	− 13.6	− 8.6	− 7.5	− 11.8	− 8.8	− 9.0	Tetradifon	− 1.7	− 2.7	− 4.8	− 4.4	− 11.3	− 8.2	− 6.1
Fenthion-oxon-sulfone	− 2.9	6.8	− 13.1	− 8.6	− 13.8	− 12.6	− 12.0	Vinclozolin	− 7.7	− 7.3	− 9.0	− 6.3	− 13.5	− 9.4	− 8.7
Fenthion-oxon-sulfoxide	15.3	30.8	16.8	20.9	− 0.1	6.3	5.8								

The recovery rate of the sample solution with acetone was calculated, at each concentration, and the difference from 100% was taken as the matrix effect values

The recovery rates of fenchlorphos oxon and paraoxon-methyl did not decrease in the recovery test in DKT without l-gulonic acid γ-lactone (Table 2), and it decreased in that with l-gulonic acid γ-lactone (Table 4). Moreover, the value of the matrix effect was low, at all concentrations (Table 5). From the above, it is suspected that the combination of the components in DKT and l-gulonic acid γ-lactone could have affected the GC–MS/MS analysis and decreased the recovery rate.

The recovery rate of profenophos decreased, even when l-gulonic acid γ-lactone was not utilized. Therefore, we presumed that l-gulonic acid γ-lactone was not the cause of the decrease in the recovery rate of profenophos, and the matrices, which originated from DKT, affected the GC–MS/MS analysis.

For cyhalothrin, deltamethrin, delta-BHC, and dicofol, the recovery rates were less than 70%, or more than 120%, in the evaluation of the purification column without the matrix effect inhibitor, although it was within the range with the matrix effect inhibitor. It is known that cyhalothrin and deltamethrin are transformed into their respective isomers by heat during GC analysis [21]. Dicofol is known to be decomposed by heat during GC analysis, and its decomposition product, 4,4′-dichlorobenzophenone, was the subject of analysis [22]. Furthermore, it was inferred that l-gulonic acid γ-lactone maintained the constant ratio of the isomers or decomposition products in the acetone and DKT sample solutions. For the delta-BHC, it was inferred that the recovery rate was recuperated because GC analysis was stabilized by the influence of l-gulonic acid γ-lactone.

For bromopropylate, fensulfothion, fensulfothion oxon, fensulfothion-oxon-sulfone, fenthion-oxon-sulfoxide, and fenthion sulfoxide, cyhalothrin, deltamethrin, delta-BHC, and dicofol, it may be challenging to guarantee the quality of the performance of the analysis method when the test environment and the operators change. When performing routine tests, it may be necessary to be extra cautious and confirm its performance on a periodic basis [17]. It is very likely that the recovery rates of these pesticides would become abnormal values when the method is applied to other herbal medicines other than DKT. Therefore, particular attention should be given to the development of analysis methods for other herbal medicines other for DKT. Further, for the 16 pesticides (acephate, bromide, dichlofluanid, dichlorvos, dimethoate, omethoate, dithiocarbamates, methamidophos, monocrotophos, piperonyl butoxide, cinerin I, cinerin II, jasmolin I, jasmolin II, pyrethrin I, and pyrethrin II) that were not analyzed in this study, it is necessary to consider alternative analysis methods other than GC–MS/MS in view of the chemical properties of these compounds.

## Conclusion

Here, we developed an analytical method for testing 91 USP-listed compounds, employing DKT as the subject. The method could extract pesticides from DKT with acetone, elute them from a SepPak C18 (5 g) column with acetonitrile, elute them from a DSC-NH_2_ (2 g) column with ethyl acetate, and perform simultaneous analysis by GC–MS/MS. This analytical method was validated according to USP and proved that 88 compounds were quantifiable. The analytical method developed in this study could enable the analysis of the metabolites of organophosphorus pesticides, which had not been analyzed and increase the number of compounds that could be analyzed.

## Abbreviations

DKT: Daikenchuto
EI: Electron ionization
GC–MS/MS: Gas chromatography–tandem mass spectrometry
HPLC: High-performance liquid chromatography
MRL: Maximum residue limit
PCB: Polychlorinated biphenyl
RSD: Relative standard deviation
